# Major Metabolites and Microbial Community of Fermented Black Glutinous Rice Wine With Different Starters

**DOI:** 10.3389/fmicb.2020.00593

**Published:** 2020-04-17

**Authors:** Li Jiang, Wei Su, Yingchun Mu, Yu Mu

**Affiliations:** ^1^School of Liquor and Food Engineering, Guizhou University, Guiyang, China; ^2^Guizhou Key Laboratory for Fermentation Engineering and Biopharmaceuticals, Guizhou University, Guiyang, China

**Keywords:** black glutinous rice wine, microbial diversity, high-throughput sequencing, flavor, correlation

## Abstract

Black glutinous rice wine (BGRW) is a traditional Chinese rice wine that is brewed using multiple strains. However, the roles of these microorganisms, particularly their contributions to aroma formation, are poorly understood. Accordingly, the main goal of this study was to determine the microbial communities and major metabolites of different traditional fermentation starters. Anshun (AS) starter and Xingyi (XY) starter were used for BGRW to provide insight into their potential contributions to the variation in flavor and aroma. High-throughput sequencing of the microbial community using the Illumina MiSeq platform revealed significant differences during fermentation between the two starter groups. *Pediococcus*, *Leuconostoc*, and *Bacillus* were the dominant bacterial genera in the AS group, whereas *Leuconostoc*, *Pediococcus*, and *Gluconobacter* were the dominant genera in the XY group. In addition, *Rhizopus*, *Saccharomyces*, and *Saccharomycopsis* were the predominant fungal genera detected in both samples. The major metabolites in the two groups were identified by high-performance liquid chromatography and headspace-solid-phase microextraction gas chromatography–mass spectrometry. A total of seven organic acids along with 47 (AS) and 43 (XY) volatile metabolites were detected, among which lactic acid was the primary organic acid, and esters were the largest group in both types of wine. Principal components analysis further revealed significant differences in the dynamic succession of metabolites between the two samples. Correlation analysis showed that 22 and 17 microorganisms were strongly correlated with the production of major metabolites in AS and XY, respectively. Among them, *Pediococcus*, *Leuconostoc*, *Lactobacillus*, *Lactococcu*s, and *Streptococcus* were shown to play crucial roles in metabolite synthesis. Overall, this study can provide a valuable resource for the further development and utilization of starters to improve the aromatic quality of BGRW.

## Introduction

Guizhou black glutinous rice wine (BGRW) is a traditional Chinese rice wine that is distinguished by the use of the local specialty black glutinous rice as the raw material, conferring unique characteristics to flavors and tastes. Black glutinous rice (*Oryza sativa L*), as one of the most popular varieties of brown rice, has been called “black pearl,” owing to its high content of phenols, flavonoids, and especially anthocyanins (Shen et al., 2009). Anthocyanins are antioxidants with nutritive properties that can help suppress the rate of cell damage and thus prevent carcinogenesis (Hou, 2003; Fimognari et al., 2008). Hence, consumption of black rice or black rice products can increase cell-defensive actions and prevent reactive oxygen species–induced diseases (Junka et al., 2016). Black glutinous rice wine is very popular in the Chinese market because the nutrients in the raw materials are dissolved in the wine, resulting in a product that is enriched in protein, amino acids, and multivitamins (Zhao, 2016). In addition, BGRW has many potential medicinal properties, including antioxidant ability and a protective effect on the kidney (Su et al., 2015; Qi et al., 2018). Thus, the use of black glutinous rice as a raw material not only can provide a variety of rice wines that are enriched in nutrients with benefits for physiological activities but also can meet the increasing consumer demands for healthier products.

The traditional BGRW brewing process involves inoculation of a starter in the glutinous rice, saccharification in an open environment, and subsequent aging for fermentation. The Anshun (AS) starter and Xingyi (XY) starter in the Guizhou province were fermented by *Rhizopus* inoculated from rice meal and herbs, with relatively high saccharification and liquefaction capabilities, and high wine yield, making them the first choice for traditional BGRW fermentation. For AS starter, white peony (*Paeonia suffruticosa* Andr., Paeoniaceae), hizoma chuanxiong (*Ligusticum chuanxiong* Hort., umbelliferae), and asarum (*Asarum sieboldii* Miq., Aristolochiaceae) were used. For XY starter, white peony (*P. suffruticosa* Andr., Paeoniaceae), mint (*Mentha haplocalyx* Briq., Labiatae), mulberry leaf (*Morus alba L.*, Moraceae), and common vladimiria root (*Aucklandia lappa* Decne., Compositae) were used. The microbial species, along with their relative abundances and interactions, in the starter cultures play an important role in fermentation, which determines the ultimate productivity and flavor quality of the rice wine (Hong et al., 2016; Yang et al., 2017a). For instance, mold and some bacteria can produce liquefaction and saccharification enzymes, which hydrolyze the starch in the raw material to produce the fermentable sugars that the yeast consume to produce alcohol as a by-product (Cai et al., 2018). In addition, mold, yeast, and bacteria produce a variety of enzymes required for cellular metabolism that generates diverse small molecules, which further contribute to the flavor and aroma of the final products (Yang et al., 2017a). Therefore, it is of great significance to analyze the microflora and their major metabolites, along with their relationships, as a basis for improving the flavor quality of BGRW. Indeed, extensive research has been conducted to unravel the composition and dynamics of the microbial communities associated with Chinese rice wine (Lv et al., 2012; Tang et al., 2019). Most of these studies have been based on traditional molecular methods such as culture-dependent methods and culture-independent polymerase chain reaction (PCR)–denaturing gradient gel electrophoresis techniques (Lv et al., 2015, 2017; Liu et al., 2017). However, these methods have certain limitations in comprehensively reflecting the actual microbial diversity in a sample due to their low flux and low sensitivity.

As an alternative approach, the continuous development of high-throughput sequencing (HTS) technology now enables more in-depth and precise evaluations of complex microflora with a reasonably low cost and in a relatively short period (Delgado et al., 2014; Lee et al., 2016) and has thus been widely applied for comprehensive analyses of microorganisms in various fermented foods, such as Shaoxing rice wine (Nie et al., 2015), *Fagopyrum tataricum* (buckwheat) wine (Ren et al., 2019), and Sake (Atsushi et al., 2018). High-throughput sequencing–based analysis of bacterial 16S rRNA genes and fungal ITS genes showed correlations between the final rice wine quality and the microbial composition of the starter. However, HTS has not been used to study the dynamics of the microbial community during BGRW fermentation. Although previous studies on BGRW have focused on the volatile metabolites present (Su et al., 2017, 2018), the effect of microorganisms on the formation of these metabolites during fermentation has not been evaluated to date.

Therefore, the aim of the present study was to perform a more in-depth and accurate evaluation of the complex microbial community dynamics during the brewing process of BGRW. In particular, we attempted to provide detailed insight of the microflora of BGRW fermented using different starters based on identification with HTS technology, along with their associated organic acids and volatile compounds determined by high-performance liquid chromatography (HPLC) and headspace-solid-phase microextraction (HS-SPME) combined with gas chromatography–mass spectrometry (GC-MS). Finally, the potential correlations between the metabolites and microbiota were uncovered through Pearson correlation analysis. These results can provide new fundamental insight into the complex dynamics of microbial communities during BRGW fermentation, along with offering a reference for the technical optimization of BGRW production.

## Materials and Methods

### Sample Preparation and Collection

The starters (AS and XY) used in this study were obtained from the cities of AS and XY of Guizhou Province, China. Black glutinous rice wine was brewed in a well-known local winery following conventional procedures. In brief, 10-kg black glutinous rice was washed and soaked in water overnight at room temperature (12–19°C) and then steamed at 100°C for 1 h. After the steamed rice was cooled using cold water, it was mixed with the starters and transferred to wine jars (80 L). The amount of AS sample added was 5%, whereas 2% XY starter was added, reflecting their respective optimal growth rates. At the end of saccharification (2 days), sterile water (30 L) was added for a 24-day traditional fermentation step at 25–30°C. Triplicate independent brewing was conducted for each starter.

At 0, 2, 4, 6, 11, 17, and 24 days during fermentation, 10 g of the fermented glutinous rice was collected at random under aseptic conditions for DNA extraction and HTS. In addition, 500 mL of the fermentation mash was collected at different brewing phases under aseptic conditions to identify the organic acids and volatile compounds with HPLC and HS-SPME–GC-MS. All samples were stored at −80°C until analysis.

### DNA Purification and PCR Amplification

Total genomic DNAs were extracted from samples using the Power Soil DNA Isolation Kit (MO BIO Laboratories, Carlsbad, CA, United States) according to the manufacturer’s protocol. DNA quality and quantity were assessed by the ratios of 260/280 nm and 260/230 nm. Then, DNA was stored at −80°C until further processing. The V3–V4 region of the bacteria 16S rRNA gene was amplified with primers 338F (5′-ACTCCTACGGGAGGCAGCAG-3′) and 806R (5′-GGACTACHVGGGTWTCTAAT-3′) (Zhang et al., 2015), and the ITS1 region of the fungi was amplified with the forward primer ITS1F (5′-CTTGGTCATTTAGAGGAAGTAA-3′) and the reverse primer ITS1R (5′-GCTGCGTTCTTCATCGATGC-3′) (Buée et al., 2009).

Polymerase chain reaction amplification was carried out using as previously described (Miya et al., 2015). A total volume of 50 μL, which contained 10 μL buffer, 0.2 μL Q5 high-fidelity DNA polymerase, 10 μL high GC enhancer, 1 μL dNTP, 10 μM of each primer, and 60 ng genomic DNA. Thermal cycling conditions were as follows: an initial denaturation at 95°C for 5 min, followed by 15 cycles at 95°C for 1 min, 50°C for 1 min, and 72°C for 1 min, with a final extension at 72°C for 7 min. The PCR products from the first-step PCR were purified through VAHTSTM DNA Clean Beads (Vazyme Biotech Co., Ltd., Nanjing, China). A second-round PCR was then performed in a 40 μL reaction, which contained 20 μL 2 × Phμsion HF MM, 8 μL ddH_2_O, 10 μM of each primer, and 10 μL PCR products from the first step. Thermal cycling conditions were as follows: an initial denaturation at 98°C for 30 s, followed by 10 cycles at 98°C for 10s, 65°C for 30 s min, and 72°C for 30 s, with a final extension at 72°C for 5 min. Successful PCR amplification was verified by 1.8% agarose gel electrophoresis. All of PCR products were pooled, purified by gel extraction, and quantified using the AxyPrepDNA gel extraction kit (Axygen Corporation, Union City, CA, US) and the QuantiFluor^TM^-ST blue fluorescence quantitative system (Promega Corporation, Madison, Wisconsin, US). The purified PCR products were then mixed at equimolar ratios for sequencing on an Illumina HiSeq PE150 system (Illumina Corporation, San Diego, CA, United States) by Biomarker Technologies Co., Ltd (Beijing, China).

### Physiochemical Properties Determination

Physical and chemical indexes such as total sugar, alcohol, total acid, and amino nitrogen were tested according to Chinese national standard (GB/T 13662, 2018).

### Determination of Flavor Compounds

#### Organic Acids Analysis

The analysis of organic acids in the samples was performed by HPLC, according to Yu (2014), with some modifications. A 5-mL sample in a tube was centrifuged at 10,000 revolutions/min for 10 min and filtrated through a 0.45-μm microporous membrane. The separations were carried out using an Agilent 1260 Infinity II system (Agilent Technologies Inc., Palo Alto, CA, US), equipped with a 4.6 × 150-mm and a 5-μm welch ultimate Zorbax SB-Aq column. The column temperature was set at 30°C. A mixture of phosphate buffer (0.02 mol/L Na_2_PO_4_), adjusted with 5% (wt/vol) phosphate solution to pH (2.70), was used as the mobile phase. The flow rate was 1 mL/min (1–4 min), 0.3 mL/min (4–10 min), and finally 1 mL/min after 10 min. The detection wavelength was 210 nm.

#### Volatile Compounds Analysis

Volatile compounds in the BGRW samples were analyzed by HS-SPME–GC-MS following the method described by Yang et al. (2017a), with modifications. Each sample (5 mL) was placed in a 20-mL SPME glass vial together with 1 g of sodium chloride and 10 μL of the internal standard 2-octanol (40.34 mg/L in absolute ethanol). The fiber was headed into the SPME device, which was inserted in the vial and shaken at 50°C for 30 min to extract and absorb the volatile compounds. Then, it was desorbed in 7 min at 250°C into the GC inlet with the automatic autosampler. Volatiles analysis was carried out using a Trace GC Ultra gas chromatograph-DSQ II mass spectrometer (Thermo Electron Corp., Waltham, MA, United States) equipped with an DB-Wax column (60 m × 0.25 mm × 0.25 mm; Agilent Technologies) and a flame ionization detector, attached to a mass spectrometer. The GC operation condition was as follows: an inlet temperature of 250°C, split ratio of 10:1, and helium (purity: 99.999%) carrier gas flow of 1 mL/min. The oven temperature program was as follows: 40°C (0 min), 4°C/min to 150°C (5 min), 3°C/min to 200°C (6 min), and finally 3°C/min to 230°C (6 min). The ion energy for the electron impact was kept at 70 eV. The chromatograms were recorded by monitoring the total ion currents in the 30–350 mass range. The constituents were tentatively identified by matching the mass spectrum with the NIST5 spectrum database and verified by comparison of their Kováts retention indices (RIs) with the RI reported in literatures, calculated using C7-C40 n-alkanes.

### HTS and Sequence Analysis

Raw sequencing data obtained from the Illumina platform were spliced using FLASH software (version 1.2.11) (Magoc and Salzberg, 2011) and filtered with Trimmomatic software (version 0.33). Finally, the UCHIME tool in QIIME software was used to remove any chimeras and retain only high-quality tag sequences (Caporaso et al., 2010) (version 8.1). The UCLUST tool in QIIME software (version 1.8.0) was further used to cluster the tags at a 97% similarity level to obtain operational taxonomic units (OTUs), and taxonomic annotation was carried out on the obtained OTUs based on the Silva (bacteria) and UNITE (fungi) taxonomic databases (Edgar et al., 2011). The sample alpha-diversity indices were evaluated using MOTHUR software (version v.1.30). The relative abundances of the representative taxa were visualized with a columnar graph using EXCEL 2013 software (Microsoft Office, Redmond, Washington, US). Principal coordinate analysis (PCoA) was performed using QIIME software.

### Statistical Analysis

Statistical analysis was performed using SPSS 17.0 (SPSS Inc., Chicago, IL, United States). The data obtained were subjected to one-way analysis of variance, and the significance level was considered at *P* < 0.05. The organic acid data were mainly performed using Origin (version 2018) (Origin Lab Inc., Hampton, MS, United States). Principal component analysis (PCA) was performed to analyze the profiles of the volatile compounds using SIMCA software (version 14.1) (UMETRICS, Sweden). Pearson correlation was used to examine the correlation between microbial genera and major metabolites, and the correlation networks between the selected flavor metabolites and microbial community were visualized via the Cytoscape software (version 3.5.1).^1^ All experiments were completed in triplicates and data were expressed as means ± standard deviations.

## Results

### Comparison of Microbial Community Diversity Among Samples

The change in the microbial composition during BGRW fermentation with the two different starters was investigated by Illumina HiSeq sequencing analyses. The numbers of effective bacterial and fungi sequences were in the range of 66,411 to 70,493.33 and 72,397.33 to 77,216.67 for AS, while 608,87 to 70,812.51 and 72,608 to 76,985.67 for XY, respectively (Supplementary Table S1). Fungal sequences greatly outnumbered those of bacteria in all samples; however, the total number of bacterial OTUs was far greater than that of fungi (Supplementary Table S1). In addition, the rarefaction curve based on OTUs approached the saturation plateau (Supplementary Figure S1), suggesting that the sequencing depth was adequate to represent the actual microbial structure of the samples. Dynamic changes to alpha-diversity indices during BGRW fermentation showed that richness and diversity of two samples were changed significantly during the fermentation process (Supplementary Table S2). Based on the 16S rRNA and ITS gene amplicons, Unifrac distance-based weighted PCoA was conducted to evaluate similarities and differences in the microbiota among the different samples. For the bacterial communities, clustered corresponding to the AS samples could be observed from 11 to 24 days of fermentation and to the XY samples could be observed from 6 to 24 days (Supplementary Figures S2A,B). For the fungal communities, clustered corresponding to the AS and XY samples could be observed from 2 to 24 days of fermentation (Supplementary Figures S2C,D).

### Microbial Community Dynamics During BGRW Fermentation

**FIGURE 1:**
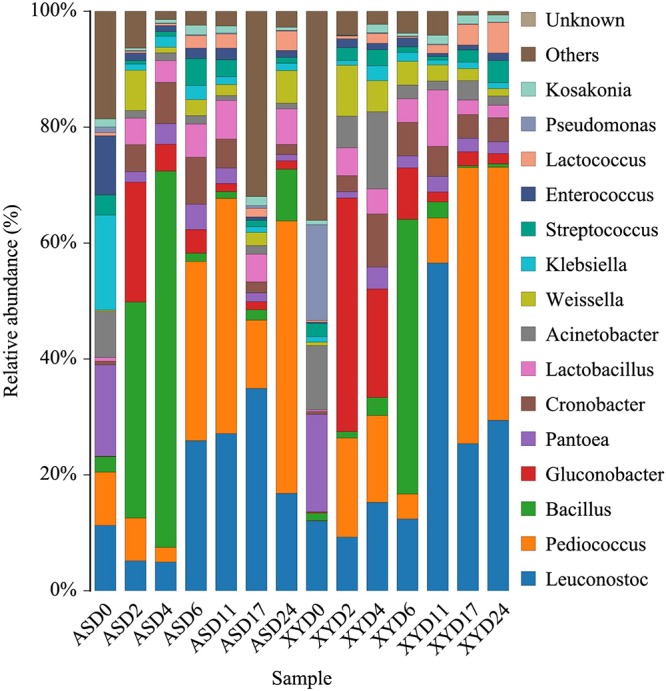
Relative abundance of bacterial genera during different fermentation stages of black glutinous rice wine with two kinds of starters.

Classification of 16S rRNA gene sequencing reads revealed 118 bacterial genera in the two samples. Microorganisms with a relative abundance greater than 1% were defined as core microorganisms, and their changes during fermentation are shown in Figure 1. With respect to the overall proportion, *Pediococcus*, *Leuconostoc*, and *Bacillus* were the dominant genera in AS, whereas *Leuconostoc*, *Pediococcus*, and *Gluconobacter* were the predominant genera detected in XY. At day 0, *Pantoea* (19.27%) and *Klebsiella* (14.35%) occupied the main position in AS samples, whereas *Leuconostoc* (12.40%) was the predominant bacterial genus in the XY samples. From days 2–4 of fermentation, the microbial community of the AS samples was characterized by high ratios of *Bacillus* (37.10 and 67.10%), whereas XY was characterized by *Gluconobacter* (40.34 and 18.75%). From day 6 to 24, *Pediococcus* and *Leuconostoc* were the prevailing genera in AS. However, in XY, the proportion of *Bacillus* increased to the highest level of 64.09% at day 6 and then showed a subsequent rapid decline in parallel with the increase in abundance of *Leuconostoc* and *Pediococcus*.

**FIGURE 2:**
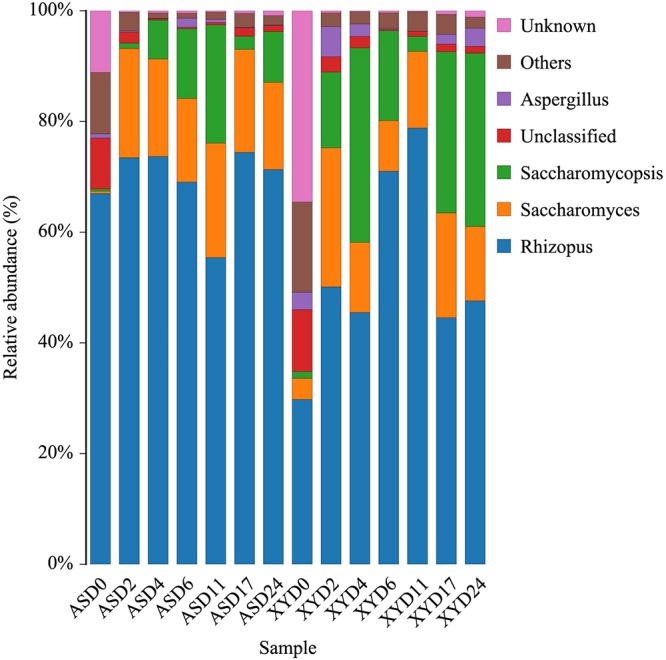
Relative abundance of fungal genera during different fermentation stages of black glutinous rice wine with two kinds of starters.

The classification of ITS gene sequencing reads detected 52 fungal genera for the two samples. As shown in Figure 2, *Rhizopus*, *Saccharomyces*, *Saccharomycopsis*, and *Aspergillus* were identified as the core fungal genera in both wines. Nevertheless, some differences in the fungal communities were observed between AS and XY during the brewing process. The overall relative abundance of *Rhizopus* in AS was greater than that in XY, and this advantage was maintained until the end of fermentation. In XY, the relative abundance of *Rhizopus* dramatically increased from an initial value of 29.79 to 78.81% detected on day 11 of fermentation, followed by a subsequent rapid decrease to 46.61%. However, the change in yeast strains (*Saccharomyces*, *Saccharomycopsis*) showed an opposite trend; thus, the increase in *Saccharomyces* and *Saccharomycopsis* was parallel to the decline in the abundance of *Rhizopus* in both starter groups. The overall proportion of *Aspergillus* in XY was higher than that in AS, with the highest proportion (0.05%) detected at day 2.

### Dynamics of Physicochemical Properties During BGRW Fermentation

**FIGURE 3:**
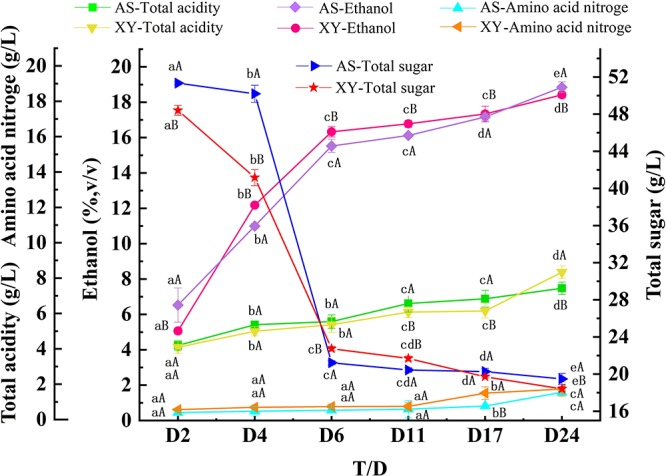
Changes in physicochemical properties during fermentation of black glutinous rice wine using two different kinds of starters (*n* = 3). **a**–**e:** Different letters represent significant differences (*p* < 0.05) during fermentation. **A,B:** Different letters represent significant differences (*p* < 0.05) in two samples.

The physicochemical properties of the AS and XY samples during the different brewing stages (including total sugars, total acids, alcohol, and amino acid nitrogen) are summarized in Figure 3. Although the fermentation performance of the two samples showed similar trends during fermentation, differences between AS and XY were still observed. During fermentation, the total sugar concentration of the BGRW continuously diminished from an initial concentration of 51.34 g/L to a final concentration of 19.72 g/L in AS and from 48.41 to 18.48 g/L in XY. Similarly, the alcohol content gradually increased from 5.07 to 18.42 vol/vol% in AS and from 6.53 to 18.85 vol/vol% in XY. According to the results of the microbial community summarized above, *Rhizopus* was dominant during the fermentation process, indicating that the glucoamylase produced by *Rhizopus* hydrolyzes starch into fermentable sugars for the yeast to convert into ethanol. Acids are immediately produced as fermentation proceeds. Indeed, the total acid content progressively increased during fermentation, and both samples showed the highest total acid content at the end fermentation, demonstrating that the acid-producing strains were most active in the later fermentation stage; however, XY (8.39 g/L) showed a slightly higher final acid content than AS (7.48 g/L). In addition, the content of amino acid nitrogen in the two wines showed an increasing trend, although in this case, the final acid content was greater in AS (1.60 g/L) than in XY (1.58 g/L) at the end of fermentation.

### Dynamics of Organic Acids and Volatile Compounds During BGRW Fermentation

**FIGURE 4:**
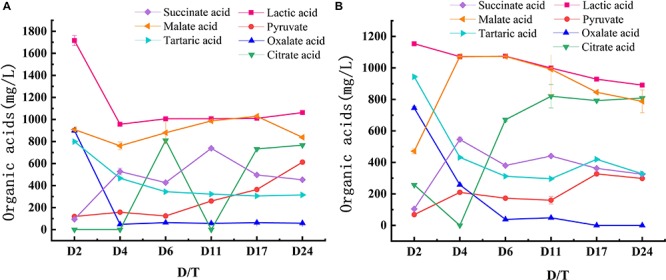
Changes of organic acids content during fermentation of black glutinous rice wine with two kinds of starters. **(A)** AS and **(B)** XY.

During fermentation, organic acids react with alcohols to generate esters, which are the main aroma compounds that can buffer and adjust the aroma and taste as well as improve the acidity of wine. Seven organic acids were detected in the two wines: pyruvate, lactic, oxalic, citric, succinic, malic, and tartaric acids. Figure 4 shows the trends of organic acids production during fermentation. Lactic acid was the primary acid detected, followed by malic acid. The lactic acid level in XY was significantly (*P* < 0.05) higher than that in AS, which showed a downward trend in both samples. However, the change of malic acid content in the two samples differed. The malic acid content in AS increased to the highest point at day 6 and then subsequently decreased until the end of fermentation. By contrast, in XY, the malic acid content declined at 4 and 24 days but showed an upward trend from 6 to 17 days. The content of oxalic acid and tartaric acid showed a decreasing trend in both samples, but the tartaric acid content in AS increased slightly at 17 days and then decreased thereafter. In addition, the levels of pyruvate and succinic acids both showed an upward trend. Compared with AS, the content of citric acid in XY fluctuated greatly, and the difference between the two samples was statistically significant (*P* < 0.05).

**FIGURE 5:**
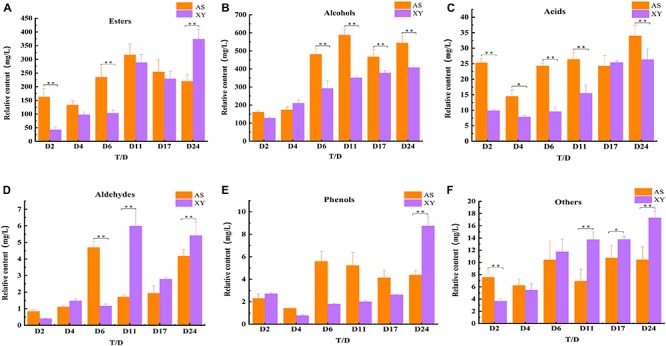
Changes in alcohols **(A)**, esters **(B)**, acids **(C)**, aldehydes **(D)**, phenols **(E)**, and others **(F)** during fermentation of black glutinous rice wine with AS or XY starter. (*n* = 3, *0.01 < *p* < 0.05, **p < 0.01).

Forty-six volatile compounds were identified in AS, including 20 esters, 10 alcohols, 5 acids, 3 aldehydes, 3 phenols, and 5 others (alkanes, ketones, alkenes). Forty-three of these volatile compounds were detected in XY, including 20 esters, 9 alcohols, 3 acids, 3 aldehydes, 3 phenols, and 5 others (alkanes, ketones, alkenes). Although no significant (*P* > 0.05) differences in the type and amount of volatile compounds were observed between AS and XY, significant (*P* < 0.05) differences in the content of flavor compounds were detected (Figure 5). In general, the levels of alcohols (Figure 5A) and acids (Figure 5B) in AS were higher than those in XY, and some compounds, including benzyl alcohol (C9), isobutyric acid (S2), and butyric acid (S3), were found only in AS. After fermentation, both samples had higher concentrations of ethanol (C1), 2-methyl-1 propanol (C2), isoamyl alcohol (C4), and phenylethanol (C10). Overall, esters were the largest group detected in both types of wine (Figure 5C). Esters dominated the AS samples from day 2 to 17 of fermentation but were detected at significantly higher levels (*P* < 0.05) in the XY samples at day 24. At the end of the fermentation, AS was mainly characterized by ethyl lactate (Z3), octanoic acid ethyl ester (Z4), decanoic acid ethyl ester (Z7), and octanoic acid ethyl ester (Z4), whereas XY was mainly characterized by decanoic acid ethyl ester (Z8), phenyl ethyl acetate (Z13), myristic acid ethyl ester (Z16), ethyl 9-hexadecenoate (Z18), and linoleic acid ethyl ester (Z20). The aldehydes (Figure 5D) and phenols (Figure 5E) showed fluctuating trends in the two samples. After fermentation, AS had a higher concentration of aldehydes, while XY had a higher concentration of phenols. 2-Methoxy-phenol (P1) and benzaldehyde (Q2) were detected as characteristic compounds at the end of fermentation. In addition, a small number of other compounds (such as ketones, alkenes, alkanes) were detected in the BGRW (Figure 5F).

### PCA of the Major Metabolites During Different Brewing Stages

**FIGURE 6:**
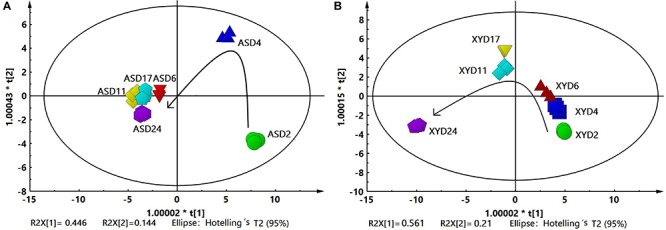
Principal components analysis scores plots of major metabolites produced during the fermentation process of black glutinous rice wine with two kinds of starters. **(A)** AS and **(B)** XY.

The dynamics of the two wines during fermentation were visualized by PCA, using the concentrations of the compounds detected in the AS and XY samples as analytical variables. Figure 6A shows the relationships of the 53 flavor compounds in AS (including 7 organic acids and 46 volatile compounds), in which the first principal component (PC1) and second principal component (PC2) explained 44.64 and 14.40% of the total variance, respectively, reflecting 61.04% of the total flavor information in AS during brewing. Figure 6B shows the close associations among the 50 flavor compounds in XY (including 7 organic acids and 43 volatile compounds), in which PC1 and PC2 explained 56.10 and 21.00% of the total variance, respectively, reflecting 67.10% of the total flavor information in XY during brewing. The PCA scores can be used to quantify the difference in the distribution of the samples at different fermentation stages, which may be caused by the different starters. The flavors in AS changed significantly (*P* < 0.05) at the early stage of fermentation (2–6 days) and clustered after 6 days of fermentation, indicating that the flavors changed slowly during 6–24 days. By contrast, the dynamics of flavor components showed an opposite trend in XY in which the samples clustered during the first 2–6 days of fermentation and then changed significantly during 6–24 days.

### Correlations Between Microorganisms and Major Metabolites

**FIGURE 7:**
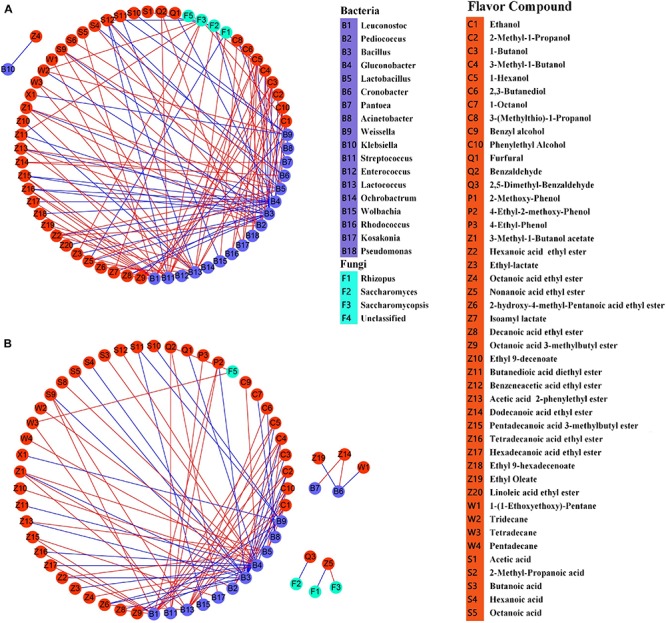
Correlation network between major metabolites and microbial communities (based on Cytoscape software) during fermentation of black glutinous rice wine with two kinds of starters. **(A)** AS and **(B)** XY. Orange circles represent the flavor metabolites; purple circles represent bacteria, and cyan circles represent fungi. The red lines represent positive correlations, and the blue lines represent negative correlations.

Finally, to determine the relationships among the metabolites and core microorganisms detected in the BGRW, correlation matrixes were constructed based on Pearson correlation coefficients. Here, we only focus on correlation coefficients showing significance at the 0.05 threshold level. The correlation coefficients showed very strong relationships in both samples (from 0.80 to 1). A total of 22 microbial genera were strongly correlated with 45 metabolites in AS (Figure 7A) and 17 microbial genera were strongly correlated with 44 metabolites in XY (Figure 7B). Key microorganisms were defined as those with at least 10 connections. A total of seven and four key microorganisms were detected in AS and XY, respectively, that affected the change of metabolites: *Gluconobacte*r, *Bacillus*, *Streptococcus*, *Lactobacillus*, *Lactococcus*, *Pediococcus*, and *Leuconostoc*. Most of these microorganisms belong to the lactic acid bacteria (LAB), indicating that LAB play a vital role in BGRW fermentation. In addition to generating lactic acid, LAB may also promote the production of other flavors, such as alcohols, esters, and alkanes (Mukisa et al., 2016). Indeed, the abundance of LAB was positively correlated with numerous esters, alcohols, acids, and alkanes. However, *Gluconobacter* and *Bacillus* were positively correlated with butanoic acid (S3), lactic acid (S6), citric acid (C9), tartaric acid (S12), benzyl alcohol (C1), octanoic acid ethyl ester (Z4), ethyl 9-decenoate (Z4), 1-(1-ethoxyethoxy)-pentane (W1), and tridecane (W2).

## Discussion

This is the first study to comprehensively compare BGRW components produced by two different starters. The results provide new insight into changes in major metabolites and the microbial community throughout the brewing process. To explore the differences between the two starter samples, a comprehensive investigation of the microbial communities in different fermentation starters for GBRW was carried out using HTS technology. The results showed that the different starters clearly influenced the microbial diversity of the BGRW. A total of 18 bacteria and 4 fungi were identified as the dominant microorganisms in the two wine types. *Pediococcus*, *Leuconostoc*, *Bacillus*, *Pantoea*, and *Lactobacillus* were the main dominant bacterial genera in AS, whereas *Leuconostoc*, *Pediococcus*, *Gluconobacter*, *Bacillus*, and *Acinetobacter* were the main dominant bacterial genera in XY. In addition, several genera of the Lactobacillales order (including *Lactococcus*, *Lactobacillus*, *Pediococcus*, *Streptococcus*, *Weissella*, *Enterococcus*, and *Leuconostoc*) were detected throughout the 24-day fermentation. The same general result was reported in Hong Qu glutinous rice wine (Liu et al., 2018). Lactic acid bacteria are ubiquitous in fermented foods because they can withstand the low pH (Abriouel et al., 2006). As functional microorganisms, LAB play a key role in the fermentation of foods, and it has been shown to improve the sensory quality of rice wine by enhancing the digestibility of protein and increasing the availability of nutrients (Luana et al., 2014). *Bacillus* was reported to be the dominant bacterium in the fermentation of rice wine (Dijl and Jong, 1993; Lv et al., 2015; Liu et al., 2018), as it has the ability to secrete a large number of enzymes to extract conutrients for the production of other microorganisms, thus promoting the formation of the rice wine flavor (Liu et al., 2008). However, in the present study, the abundance of *Bacillus* decreased in conjunction with the increase in the abundance of *Pediococcus* and *Lactococcus* (Figure 1), indicating an antagonistic relationship between these genera. Koyanagi et al. (2013) reported that *Bacillus* might produce growth factors that are conducive to LAB growth during the postfermentation stage. *Gluconobacter* is a functional bacterium in fermented vinegar that can produce acetic acid and has also been found in Grenache grape wine (Portillo and Mas, 2016) and Chinese liquor (Wang et al., 2017) as the dominant microorganism. *Gluconobacter* is usually sensitive to alcohol and can be inhibited by high alcohol concentrations (Joyeux et al., 1984; Toit and Pretorius, 2002; González et al., 2005). This explains why its prevalence was greater in the early stage of fermentation, especially in the XY samples. *Pantoea* is common endophytes of rice (Megías et al., 2016) with antibacterial properties and is therefore often used as a biological control agent. In this study, the predominant bacterial genus *Pantoea* observed at day 0 might originate from the raw material of black glutinous rice and the starters. Similarly, *Pantoea* has been identified as the most dominant bacteria in Hong Qu starters (Coutinho and Venter, 2009; Walterson et al., 2014).

In addition to bacteria, rice wine fermentation also depends on mold and yeast. In particular, *Rhizopus*, *Saccharomyces*, and *Saccharomycosis* are popular genera in starters and are considered to be important functional microorganisms. Anshun had a higher proportion of *Rhizopus*, whereas XY had a higher proportion of yeast, which may be related to the different herbs added to the starter. *Rhizopus*, a strong amylase producer and frequently found in amylolytic fermentation starters for rice wine brewing (Dung et al., 2007; Thanh et al., 2008), was reported that the main fungi in Yao Qu (Lv et al., 2012). *Saccharomyces* is the most effective ethanol producer known so far (Vaughan-Martini and Martini, 1995), whereas *Saccharomycopsis* is a non-*Saccharomyces cerevisiae* with high amylase and glycosidase activities (Charoenchai et al., 1997). It has been reported that the yeast may act as a biocontrol agent against spoilage microorganisms increasing the shelf life of the product (Bora et al., 2016). Moreover, yeasts can stimulate LAB growth by supplying essential metabolites including pyruvate, amino acids, and vitamins (Jespersen, 2003). In both samples, the relative abundance of *Rhizopus* showed opposing dynamics to *Saccharomyces* and *Saccharomycopsis* (Figure 2), which may reflect the ability of *Rhizopus* to outcompete yeast for nutrients.

The physicochemical properties of a sample are typically used to determine the fermentation state and degree and can also reflect the microbial state in the fermented mash. After saccharification, the main reaction in mash involves the action of yeasts that use fermentable sugars to produce ethanol and other products. Thus, the total sugar and alcohol concentrations in the AS and XY samples decreased and increased during fermentation, respectively (Figure 3). Establishment of a suitable environment in the early stage of fermentation causes the acid-producing microorganisms to multiply and produce acid metabolites, which leads to an increase of the total acid content. However, the growth of some acid-producing microorganisms is inhibited under higher acid and alcohol environments, resulting in a steady level of total acids during the later period of fermentation. The level of amino acid nitrogen directly affects the quality grade and overall flavor of wine. In line with a previous report showing that the content of amino acid nitrogen in rice wine is closely related to the amount of yeast and protein mass concentration in the mash (Pan et al., 2016), we observed similar trends in the dynamics of amino acid nitrogen and yeast during fermentation.

In addition to enhancing the flavor of rice wine, organic acids can also improve the intestinal tract and prevent fatigue. For example, malic acid can prevent fatigue, protect the liver, and enhance heart function (Liu et al., 2003), whereas citric acid can delay aging, eliminate fatigue, and reduce blood pressure (Feng et al., 2010). Correlation analysis showed that five and three microorganisms in AS and XY, respectively, were significantly correlated with the formation of organic acids. Lactic acid, as the primary organic acid in the two samples, can be esterified to form ethyl lactate, which is the main aromatic body of rice wine (Gao et al., 2014). Indeed, the high level of lactic acid in BGRW is associated with the abundance of LAB. Thus, a higher content of lactic acid was found in XY compared with AS, which likely reflects the larger proportion of LAB in XY. In addition, a positive correlation between lactic acid and *Cronobacter* was observed. Notably, *Cronobacter* is a foodborne pathogen, most commonly detected in infant formula. Fortunately, the BGRW brewing process includes a sterilization step (80°C for 30 min), which can kill most pathogenic bacteria, thereby improving the safety of rice wine. Malic acid, the second dominant organic acid, has been reported to be related to yeast and *Aspergillus* (Peleg et al., 1988; Pines et al., 1996), but no relationship was found between them. However, our study suggested that *Weissella* and *Wolbachia* were correlated significantly with malic acid, in which *Weissella* was identified as one of the dominant LAB in Dombea, a Cambodian traditional stater (Sokny et al., 2018). But its abundance begins to decrease after 2 days in two samples. In AS, *Lactobacillus*, *Pediococcus*, and *Lactococcus* were all positively correlated with citric acid, and *Bacillus* contributed to an increase in tartaric acid. Among these genera, *Lactococcus* produces acids by using carbohydrates, *Pediococcus* produces acids by fermenting sugars (Ren et al., 2019), and *Bacillus* has strong enzymatic activity and can produce abundant metabolites to synthesize various organic acids. *Gluconobacter* was reported that can promote the formation of acids, alcohols, and esters in rice wine fermentation (Saichana et al., 2014). In this study, *Gluconobacter* was positively correlated with butanoic, oxalic, and tartaric acids in XY. Because *Gluconobacter* is inhibited by higher alcohol levels (Joyeux et al., 1984; Toit and Pretorius, 2002; González et al., 2005), an increase in alcohol content during fermentation also affected the butanoic, oxalic, and tartaric acids content.

There were minimal differences in the volatile metabolites between the two starters. Correlation analysis showed that about twice the numbers of microorganisms were involved in the formation of alcohols in AS compared to those in XY. Ethanol was the most abundant alcohol detected in the two wines, which can be formed from acetaldehyde produced by LAB (Engels et al., 1997). *Lactobacillus*, *Pediococcus*, *Leucoconstoc*, *Lactococcus*, and *Saccharomycopsis* are involved in ethanol formation, all of which are LAB except for *Saccharomycopsis*. Limtong et al. (2002) reported that *Saccharomycopsis* contributes to alcohol production. Furthermore, the cooperative fermentation of *Saccharomycopsis* and *Saccharomyces* can adjust the alcohol content to a certain extent and improve the aroma quality of wine (Liu et al., 2016). The alcohols 2-methyl-1-propanol, 3-methyl-1-butanol, and phenylethyl are the metabolites of valine, leucine, and phenylalanine, respectively, and are the typical flavor compounds in fermented alcoholic beverages that also constitute the overall sensory complexity of rice wine (Chen et al., 2010; Hye-Jung et al., 2013). In AS, the level changes of 2-methyl-1-propanol, 3-methyl-1-butanol, and phenylethanol were related to the dynamics in the abundance of *Leuconostoc*, *Lactobacillus*, *Pedicoccus*, and *Streptococcus*. In XY, although the production of 2-methyl-1-propanol and phenylethanol was strongly impacted by *Leuconostoc*, none of the microorganisms detected was associated with 3-methyl-1-butanol. 2,3-Butanediol is one of the few aromatic polyols in wines, with a creamy and buttery aroma. Early studies showed that *Bacillus* in starter could produce 2,3-butanediol (Wu and Xu, 2012). We also demonstrated that LAB contributed to the formation of 2,3-butanediol, which was also detected as the characteristic compound of XY after fermentation.

Esters have been reported to endow rice wine with fruity and floral odors (Fang et al., 2015). Most of the esters detected in the present study were ethyl esters, which is in line with previous studies (Chen and Xu, 2010; Yang et al., 2017b). Ethyl ester is usually produced by the esterification of fatty acids and ethanol during fermentation and aging (Cheong et al., 2010; Yu et al., 2014), mainly due to yeast and other microorganisms metabolism (Fan and Qian, 2005). Correlation analysis indicated that *Pantoea*, *Leuconostoc*, *Lactobacillus*, *Streptococcus*, *Pediococcus*, *Enterococcus*, *Saccharomycopsis*, *Lactococcus*, *Ochrobactrum*, and *Gluconobacter* were positively correlated with esters (Figure 7). Octanoic acid, decanoic acid, and hexadecanoic acid ethyl esters were the main esters identified in this study. Decanoic acid ethyl ester has a brandy, fruity, and grape-like aroma; octanoic acid ethyl ester has a floral and pear aroma; and hexadecanoic acid ethyl ester has an apple aroma. 4-Ethylphenol and 4-ethyl 2-methoxyphenol were the main phenolic compounds detected in AS, which are the main aroma substances of red wine that are formed by the metabolism of ρ-coumaric acid and ferulic acid, respectively (Pollnitz et al., 2000). Chen and Xu (2010) also reported that 4-ethyl 2-methoxyphenol was the most abundant phenolic compound in Chinese rice wine fermented with wheat Qu with the highest odor activity value, exhibiting characteristics of a phenolic, clove, and smoke-like flavor. *Aspergillus*, *Streptococcus*, *Lactobacillus*, and *Leuconostoc* were positively correlated with 4-ethylphenol and 4-ethyl 2-methoxyphenol (Figure 7). 2-Methoxy-phenol was the characteristic phenol detected in XY, and its content was significantly higher than that of AS. This may be due to higher abundance of related microorganisms in XY than in AS. Benzaldehyde has a typical almond and cherry odor (Jung et al., 2014), and *Aspergillus* and *Streptococcus* play the greatest roles in its formation. Yoshizaki et al. (2010) stated that *Aspergillus* is related with the metabolites of aldehydes. Thus, the content of benzaldehyde was higher at the end of fermentation in XY, which may be related to the higher abundance of *Aspergillus.* Acetoin is an important physiological metabolite that is excreted by many microorganisms and serves as a quality indicator of fermented products (Xiao and Xu, 2007). Members of the genera *Acetobacter*, *Lactococcus*, *Klebsiella*, *Enterobacter*, and *Bacillus* are considered as synthetic acetoin bacteria during fermentation (Molinari et al., 2003; Chen et al., 2010). Although we did not detect a relationship between the microorganisms and acetoin, it is worth noting that correlation is not equal to causation. Overall, our results reflect the highly complex relationship between microorganisms and flavor substances during BGRW fermentation and indicate that the production of flavor substances is likely the result of the synergistic effect of microorganisms and biochemical reactions.

## Conclusion

Tracking and comparison of the physicochemical properties and microbial community structures, along with their corresponding flavors, during BGRW fermentation with two different starters showed that the physical and chemical indicators and flavors were strongly affected by the microbial community. *Pediococcus*, *Leuconostoc*, *Bacillus*, *Gluconobacter*, and *Lactobacillus* were the dominant bacteria, whereas *Rhizopus, Saccharomyces*, and *Saccharomycopsis* were the predominant fungi detected during fermentation. The potential role of microorganisms in flavor formation was also demonstrated through the Pearson correlation coefficient. Lactic acid bacteria were identified as the key microorganisms contributing to the production of flavor. These results have a certain reference value for the quality improvement and technical optimization of BGRW production. Nevertheless, further studies should be devoted to validating the association between the core microorganisms identified and the specific flavor components using multiomics approaches, including metagenomics, metaproteomics, and metatranscriptomics.

## Data Availability Statement

All sequencing data have been deposited at the Sequence Read Archive of the National Center for Biotechnology Information (NCBI), with SRA accession number: the accession number of bacteria and fungi are PRJNA597320 and PRJNA597372, respectively.

## Author Contributions

LJ and WS contributed the experimental design. LJ performed the statistical analysis and wrote the manuscript. LJ, WS, YiM, and YuM contributed to manuscript revision, read and approved the submitted version.

## Conflict of Interest

The authors declare that the research was conducted in the absence of any commercial or financial relationships that could be construed as a potential conflict of interest.

## Footnotes

1 http://www.cytoscape.org/
