# Supplementary material for: Major Metabolites and Microbial Community of Fermented Black Glutinous Rice Wine With Different Starters
Source: Front Microbiol. 2020 Apr 17;11:593. doi: 10.3389/fmicb.2020.00593 (PMC7180510; doi:10.3389/fmicb.2020.00593)
Supplement: Supplementary file 1 [file Data_Sheet_1.docx]

Supplementary Material

# Supplementary Figures and Tables

## Supplementary Figures


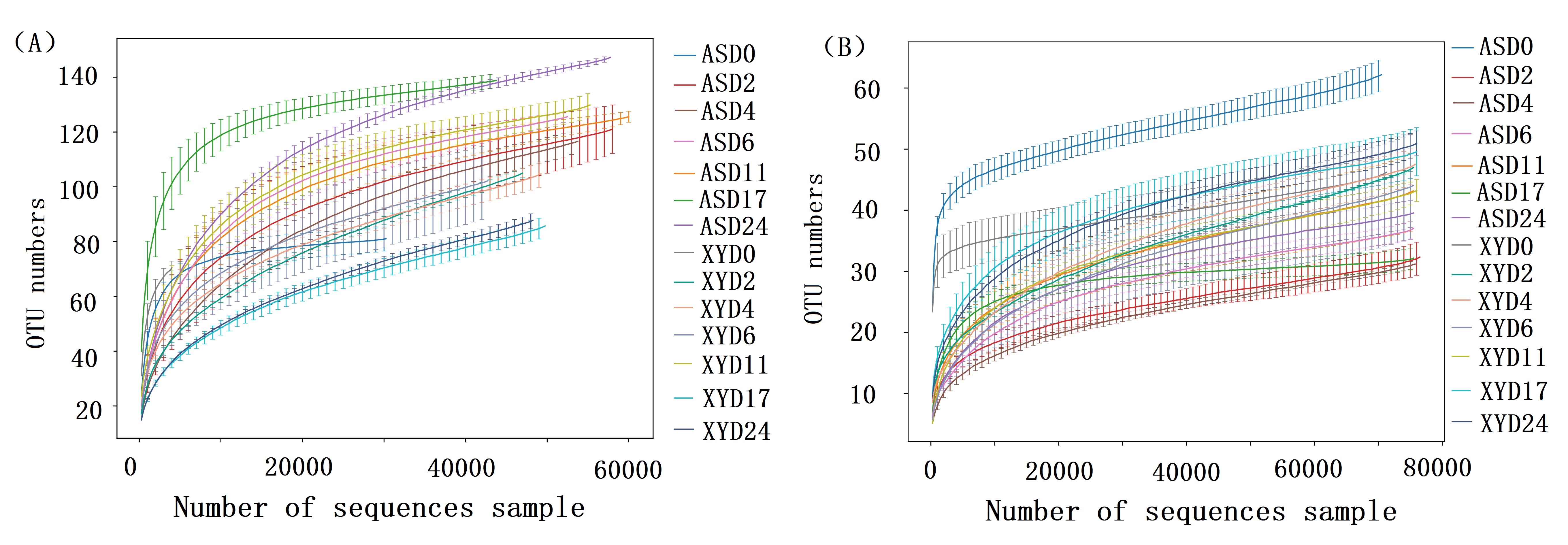


**Figure S1.** Bacteria (A) and fungi (B) rarefaction analysis of during different fermentation stages of BGRW with two kinds of starters. Rarefaction curves of OTUs clustered at 97% sequence identity for different samples.

**
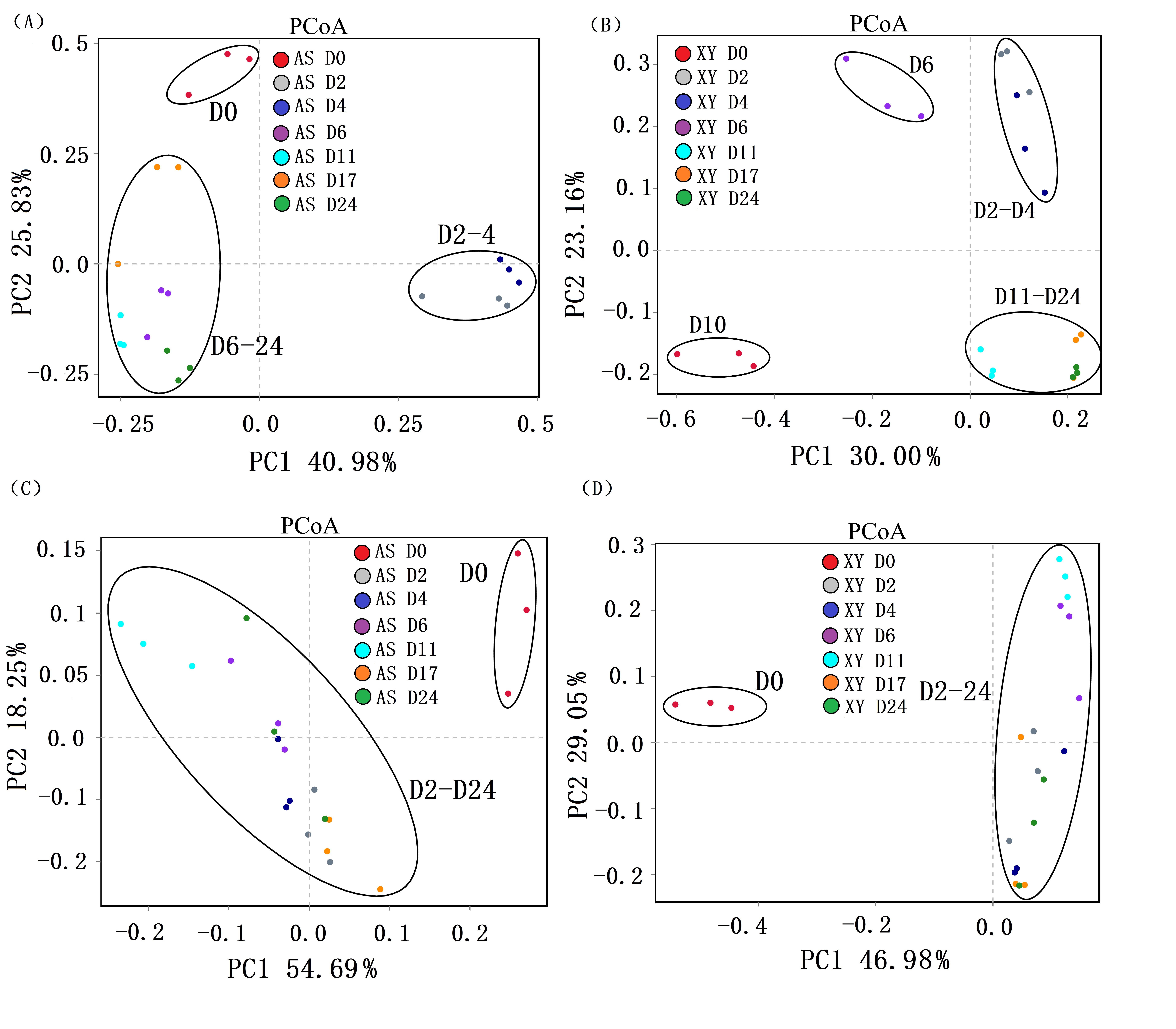
**

**Figure S2.** PCoA score plots of bacterial (A and B) and fungi (C and D ) during different fermentation stages of BGRW with two kinds of starters. (A and C) AS and (B and D ) XY.

## Supplementary Tables

**Table S1.**  OTU and sequence abundance of bacteria and fungi in different fermentation stages of BGRW with two kinds of starters.

| Sample ID | Bacteria | | | |  | Fungi | | | |
| --- | --- | --- | --- | --- | --- | --- | --- | --- | --- |
|  | AS | | XY | |  | AS | | XY | |
|  | OTU | Effective Tags | OTU | Effective Tags |  | OTU | Effective Tags | OTU | Effective Tags |
| D0 | 105.33±27.64 | 70493.33±186.78 | 127.50±5.35 | 70812.51±112.28 |  | 52.67±16.98 | 72397.33±687.09 | 41.66±4.99 | 72608.00±577.89 |
| D2 | 97.00±18.67 | 68588.00±875.45 | 122.00±11.86 | 67804.50±383.77 |  | 44.67±11.59 | 77216.67±299.05 | 47.00±5.35 | 76570.33±382.83 |
| D4 | 104.33±18.57 | 66643.00±69.23 | 132.00±7.87 | 66433.00±616.89 |  | 39.33±8.65 | 76843.00±414.95 | 44.33±8.22 | 76612.00±197.86 |
| D6 | 122.00±12.36 | 68704.67±549.70 | 89.50±5.44 | 64751.50±6546.14 |  | 34.67±1.25 | 76389.00±319.00 | 50.00±5.72 | 76447.33±233.40 |
| D11 | 145.33±4.92 | 68763.67±683.05 | 105.00±4.11 | 63502.00±342.31 |  | 35.67±6.94 | 76703.00±293.20 | 47.66±0.47 | 76751.67±270.30 |
| D17 | 127.33±16.01 | 66411.00±2169.43 | 91.50±3.68 | 60887.00±319.20 |  | 34.33±4.19 | 76628.33±320.20 | 51.33±2.87 | 76985.67±323.11 |
| D24 | 93.67±22.53 | 68390.67±448.10 | 128.00±10.96 | 65142.50±2001.38 |  | 51.67±4.99 | 76439.67±405.18 | 37.66±4.11 | 76683.33±127.04 |

Values are presented as mean ± standard error (n=3).

**Table S2.** Richness and diversity of bacteria and fungi in different fermentation stages of BGRW with two kinds of starters.

| Sample ID | Bacteria | | | |  | Fungi | | | |
| --- | --- | --- | --- | --- | --- | --- | --- | --- | --- |
|  | Shannon | | Chao1 | |  | Shannon | | Chao1 | |
|  | AS | XY | AS | XY |  | AS | XY | AS | XY |
| D0 | 2.63±0.14^Aa^ | 2.92±0.12^Aa^ | 90.33±5.19^Aa^ | 83.58±5.25^Aa^ |  | 1.62±0.10^Aa^ | 2.30±0.18^Ab^ | 77.33±10.27^Aa^ | 61.83±13.81^Aa^ |
| D2 | 2.30±0.12^ABa^ | 2.28±0.16B^Ca^ | 118.42±7.94C^Da^ | 135.12±3.80^BCa^ |  | 1.00±0.14^CDa^ | 1.58±0.06^Bb^ | 36.90±5.11^Ba^ | 58.94±4.53^Aa^ |
| D4 | 1.61±0.11^Ca^ | 2.67±0.05^Ab^ | 128.01±4.64^Ba^ | 134.94±9.19^BCb^ |  | 1.06±0.08^BCDa^ | 1.51±0.09^Bb^ | 38.26±9.33^Ba^ | 55.13±2.83^Aa^ |
| D6 | 2.48±0.12^ABa^ | 2.12±0.17^BCDb^ | 142.87±18.61^BCDa^ | 116.97±7.57^Bb^ |  | 1.23±0.07^Ba^ | 1.10±0.07^Ca^ | 40.14±3.48^Ba^ | 49.88±13.25^Aa^ |
| D11 | 3.07±0.36^Ba^ | 2.30±0.15^Ba^ | 149.95±7.47^BCa^ | 146.67±12.05^BCb^ |  | 1.44±0.07^Aa^ | 0.78±0.12^Cb^ | 58.33±25.77^ABa^ | 60.05±10.37^Aa^ |
| D17 | 2.21±0.11^Da^ | 1.94±0.15^Db^ | 133.58±2.40^CDa^ | 110.52±17.05^Bb^ |  | 0.86±0.06^Da^ | 1.56±0.08^Da^ | 32.83±2.78^Ba^ | 54.89±8.30^Ab^ |
| D24 | 2.09±0.02^Ba^ | 1.98±0.04^CDa^ | 159.40±1.38^Da^ | 117.11±8.65^Bb^ |  | 1.17±0.09^BDa^ | 1.48±0.10^Bb^ | 42.07±3.30^Ba^ | 55.43±2.31^Aa^ |

Values are presented as mean ± standard error (n=3), ^A-D^Different letters in the same column represent significant differences (*p* < 0.05), ^a-b^Different letters in the same row represent significant differences (*p* < 0.05).
